# Bullfrog farms release virulent zoospores of the frog-killing fungus into the natural environment

**DOI:** 10.1038/s41598-019-49674-0

**Published:** 2019-09-17

**Authors:** Luisa P. Ribeiro, Tamilie Carvalho, C. Guilherme Becker, Thomas S. Jenkinson, Domingos da Silva Leite, Timothy Y. James, Sasha E. Greenspan, Luís Felipe Toledo

**Affiliations:** 1 Laboratório de História Natural de Anfíbios Brasileiros (LaHNAB), Instituto de Biologia, Universidade Estadual de Campinas (UNICAMP), CEP 13083-862, Campinas, São Paulo Brazil; 2 Department of Biological Sciences, The University of Alabama, Tuscaloosa, Alabama 35487 USA; 3 Department of Ecology and Evolutionary Biology, University of Michigan, Ann Arbor, Michigan 48109 USA; 4 Departamento de Genética, Evolução, Microbiologia e Imunologia, Instituto de Biologia, Universidade Estadual de Campinas (UNICAMP), CEP 13083-862, Campinas, Sao Paulo Brazil

**Keywords:** Conservation biology, Herpetology, Fungal infection

## Abstract

Bullfrog farming and trade practices are well-established, globally distributed, and economically valuable, but pose risks for biodiversity conservation. Besides their negative impacts on native amphibian populations as an invasive species, bullfrogs play a key role in spreading the frog-killing fungus *Batrachochytrium dendrobatidis* (Bd) in the natural environment. Bullfrogs are tolerant to Bd, meaning that they can carry high infection loads without developing chytridiomycosis. To test the potential of bullfrog farms as reservoirs for diverse and virulent chytrid genotypes, we quantified Bd presence, prevalence and infection loads across approximately 1,500 farmed bullfrogs and in the water that is released from farms into the environment. We also described Bd genotypic diversity within frog farms by isolating Bd from dozens of infected tadpoles. We observed individuals infected with Bd in all sampled farms, with high prevalence (reaching 100%) and high infection loads (average 71,029 zoospore genomic equivalents). Average outflow water volume from farms was high (60,000 L/day), with Bd zoospore concentration reaching approximately 50 million zoospores/L. Because virulent pathogen strains are often selected when growing in tolerant hosts, we experimentally tested whether Bd genotypes isolated from bullfrogs are more virulent in native anuran hosts compared to genotypes isolated from native host species. We genotyped 36 Bd isolates from two genetic lineages and found that Bd genotypes cultured from bullfrogs showed similar virulence in native toads when compared to genotypes isolated from native hosts. Our results indicate that bullfrog farms can harbor high Bd genotypic diversity and virulence and may be contributing to the spread of virulent genotypes in the natural environment. We highlight the urgent need to implement Bd monitoring and mitigation strategies in bullfrog farms to aid in the conservation of native amphibians.

## Introduction

The international wildlife trade facilitates introductions of invasive species and pathogens that threaten native communities. Bullfrogs are native to eastern North America^1,2^ and are farmed on a large scale in Asia, Central America, and South America to supply the international frog leg trade^2,3^. Bullfrog farming emerged as an alternative to overharvesting native amphibian species^4^. However, this practice continues to contribute to the current global amphibian crisis by facilitating biological invasions^5–11^. Escapes of bullfrogs from farms have led to the establishment of invasive bullfrog populations^6,12–14^ that negatively influence the local anurofauna by interfering with acoustic communication and jeopardizing reproduction of native anurans^15,16^, preying on native amphibian species^17,18^ and competing for resources, thus reducing the fitness of native amphibian populations^19,20^.

Bullfrogs also play an important role in the dynamics of amphibian chytridiomycosis, a disease caused by the fungus *Batrachochytrium dendrobatidis* (Bd) that has been linked to amphibian declines worldwide^21^. Bullfrogs are highly tolerant hosts^22,23^, meaning that they are able to withstand high Bd infection loads without developing chytridiomycosis (but see exceptions in^24,25^). Thus, bullfrogs serve as competent pathogen reservoirs^12,22,26^ and international pathogen vectors^11,27,28^. Traded bullfrogs have been found to carry multiple genetic lineages of Bd, including the invasive hypervirulent global pandemic lineage (BdGPL) responsible for the decline of amphibians on several continents^11,29,30^ as well as genotypes from enzootic lineages that tend to be less virulent due to long-term coevolution with their amphibian hosts, as reported for BdASIA-2/BdBRAZIL^31,32^, BdCAPE, BdCH^29^ and BdASIA-1^11^.

The combination of bullfrog-Bd interactions and bullfrog farming practices may create ideal conditions for outbreaks of chytridiomycosis and declines of native fauna. High Bd tolerance in bullfrogs suggests that farms could function as reservoirs of particularly virulent Bd genotypes. Novel Bd genotypes introduced through trade may also have higher virulence in native hosts compared to endemic genotypes with which they have co-evolved. Furthermore, translocation of divergent Bd lineages may bring previously isolated Bd genotypes into contact, allowing for the emergence of sexually hybridized strains and possibly giving rise to more virulent hybrids than the parental lineages^28,32^. In addition, the water used in bullfrog farming is often released into the natural environment without treatment, potentially carrying viable Bd zoospores that could infect native amphibian species. Bullfrogs are also farmed in high densities, providing ideal conditions for (i) Bd transmission among hosts, (ii) hybridization to occur, and (iii) selection to operate. Thus, bullfrog farms may promote continuous spillover of pathogenic zoospores into native anuran communities and propogation of virulent Bd genotypes, but previous studies have not quantified pathogen outflow from bullfrog farms or compared the virulence of genotypes carried by farmed and native host species under controlled experimental conditions.

In Brazil, ranaculture began in the 1930s and escalated throughout the country in the 1970s^33^, coinciding with sharp historical amphibian declines throughout the Brazilian Atlantic Forest, which have been recently attributed to the emergence of Bd^34^. Recently, a Brazilian ordinance proposed that introduced aquatic species, including bullfrogs, should be considered native to foster aquaculture development^35^, which is expected to exacerbate introductions of bullfrogs into native amphibian communities. To test bullfrog farms in Brazil as a source of virulent Bd genotypes for native amphibians, we sampled Bd from farmed bullfrogs across life stages (tadpoles, juveniles and adults), as well as from the outflow water from frog farms. We also isolated and genotyped Bd strains found in farmed bullfrogs and performed infection experiments testing for differences in Bd virulence among isolates from native hosts and farmed bullfrogs. We hypothesized that because of the high Bd tolerance in bullfrogs^23,26^, isolates from bullfrogs would be more pathogenic to a native Brazilian host species than those isolated from native frogs. Combined, our results provide important information about the dynamics of Bd in the amphibian trade that should be used to guide actions directed at conserving native anurans in Brazil and elsewhere.

## Results

### Bullfrog farm assessment

**Figure 1:**
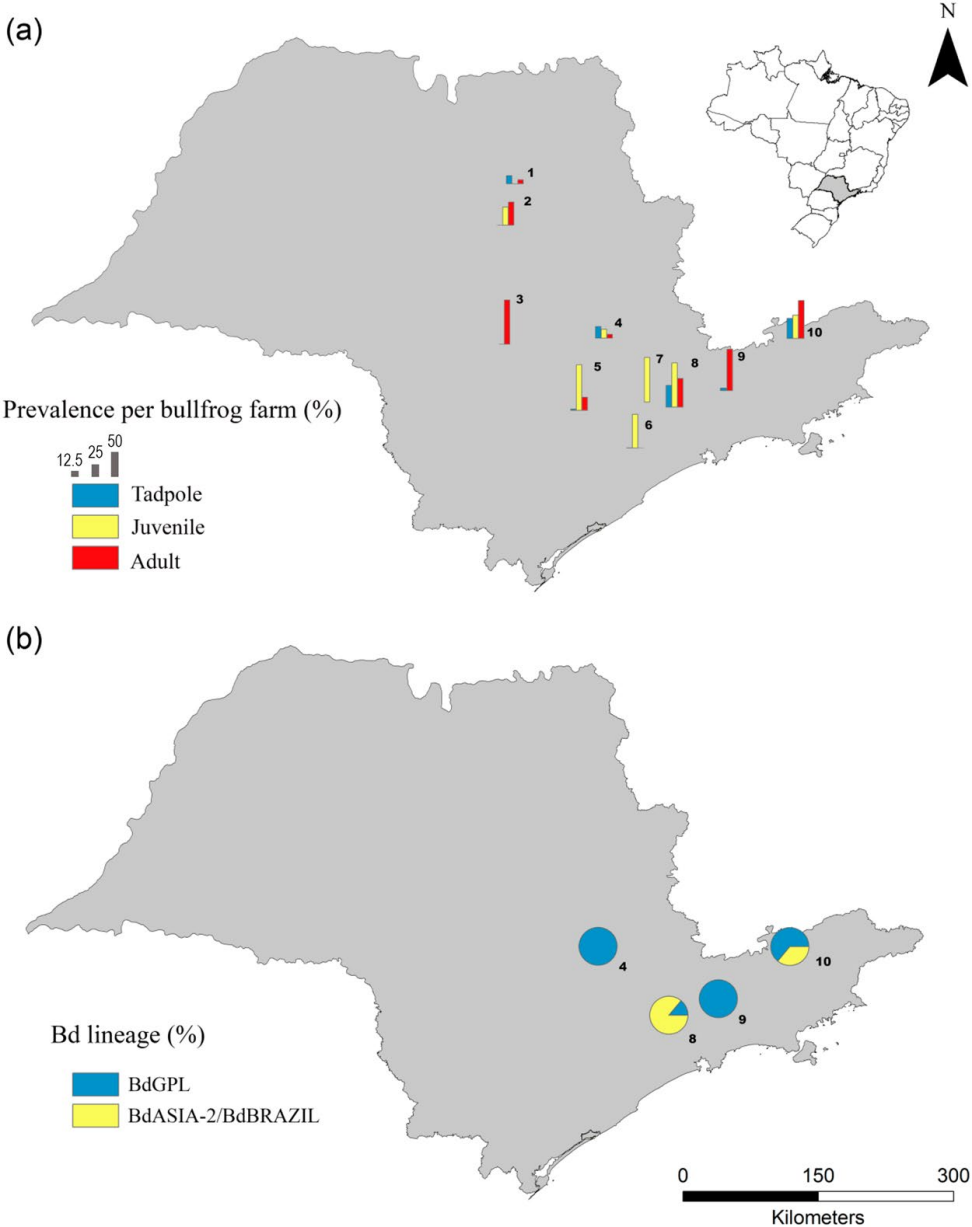
Bd prevalence in different developmental stages (tadpole, juvenile and adult) in different sampled farms (**a**); Bd lineages isolated from bullfrogs (**b**).

**Figure 2:**
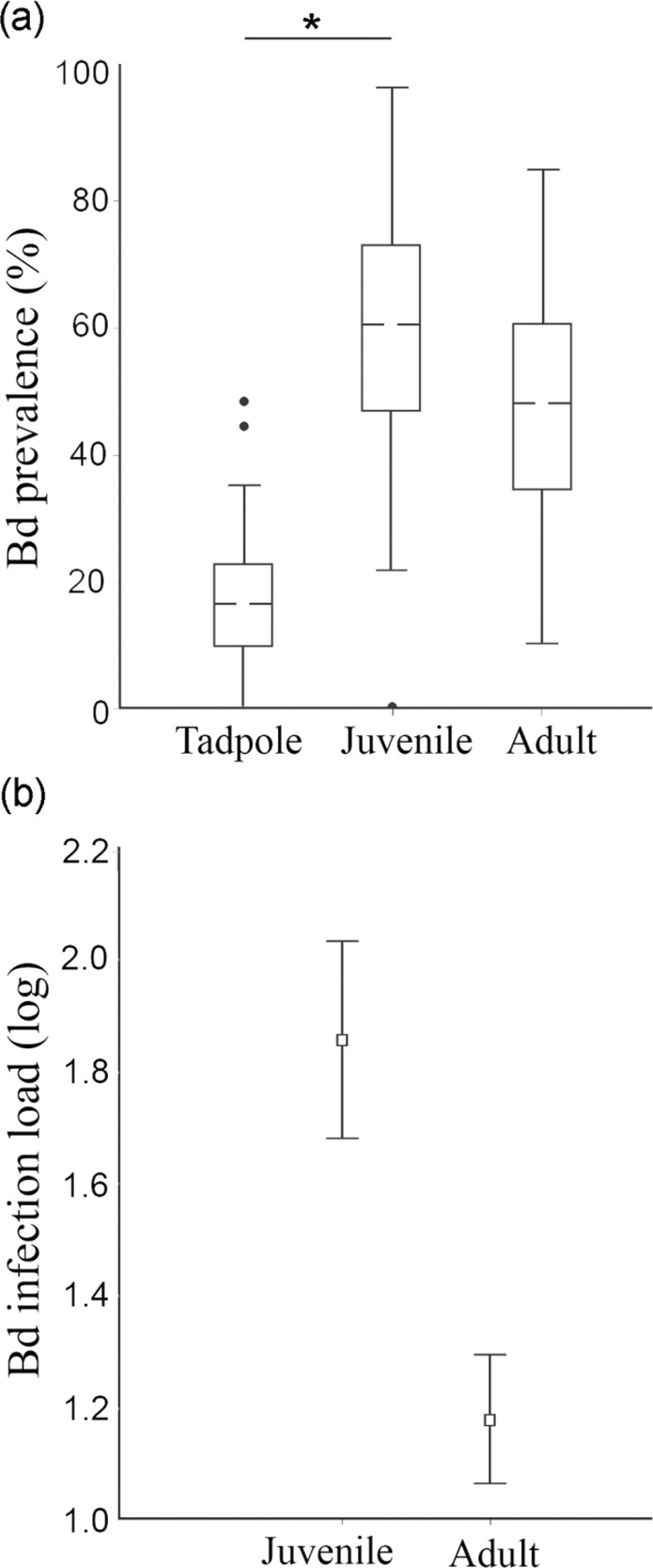
Bd prevalence (%) among the three developmental stages (tadpole, juvenile and adult) (**a**); Bd infection load by developmental stage (juvenile and adult) (**b**).

The observed Bd prevalence in farmed tadpoles ranged from 0 (2 farms) to 48%. We detected Bd^+^ juveniles at all farms, with prevalence reaching 100% and infection loads up to 71,029 zoospore genome equivalents (g.e.; Fig. 1a, Table 1). Bd prevalence varied among amphibian developmental stages (F = 4.226; *P* = 0.027), with juveniles exhibiting higher Bd prevalence than tadpoles (Tukey *P* = 0.02), but not adults (Fig. 2a). We detected the same pattern after accounting for a conservative estimate of false positive error (Supplementary Table S1, Table S2). Prevalence of Bd was similar across life stage when accounting for a conservative estimate of false negative error (Supplementary Table S1, Table S2). Juveniles presented higher infection loads than adults (*t* = 6.55, df = 1, *P* < 0.001; Fig. 2b).

**Table 1:** Developmental stage of bullfrogs, Bd prevalence [presented as percentage (Bd+/tested individuals)], and infection load [values presented as mean; SD (range)] sampled in farms.

Bullfrog farm	Stage	Prevalence (%)	Load (zoospore g.e.)
#1	Tadpole	18 (18/100)	—
	Juvenile	0 (0/35)	—
	Adult	8.6 (3/35)	4; 3 (2–7)
#2	Tadpole	0 (0/102)	—
	Juvenile	40 (14/35)	30; 48 (2–176)
	Adult	51.4 (18/35)	14; 25 (1–108)
#3	Tadpole	0 (0/100)	—
	Adult	97.2 (35/36)	255; 865 (2–5,038)
#4	Tadpole	22 (22/100)	—
	Juvenile	20 (7/35)	11; 14 (2–41)
	Adult	8.6 (3/35)	3; 3 (2–6)
#5	Tadpole	3 (3/100)	—
	Juvenile	100 (35/35)	3,095; 11,874 (35–71,030)
	Adult	28.6 (10/35)	4; 2 (2–9)
#6	Tadpole	1 (1/100)	—
	Juvenile	74.3 (26/35)	73; 162 (1–835)
	Adult	0 (0/34)	—
#7	Juvenile	98 (34/35)	94; 193 (1–1,195)
#8	Tadpole	48 (48/100)	—
	Juvenile	97.1 (34/35)	2,193; 7,208 (4–31,650)
	Adult	62.9 (22/35)	32; 53 (2–250)
#9	Tadpole	6 (6/100)	—
	Adult	91.4 (32/35)	93; 292 (3–1,597)
#10	Tadpole	44 (44/100)	—
	Juvenile	51.4 (18/35)	20; 34 (1–141)
	Adult	82.9 (29/35)	68; 144 (2–667)

**Figure 3:**
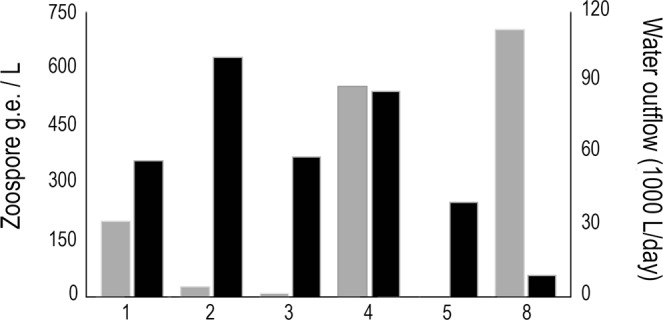
Concentration of Bd zoospores per liter of water that is released from the farms into the natural surrounding waterbodies (grey bars) and volume of outflow water released daily by the farms (black bars).

We consistently recorded high Bd zoospore concentrations in outflow water, with an average concentration of 114 Bd zoospore g.e. per liter. Outflow of water averaged 60,000 L per day, which is estimated to release approximately 50 million zoospore g.e. per day (Fig. 3).

We cultured 36 Bd isolates [7 from farm #4, 7 from farm #8, 8 from farm #9, and 14 from farm #10 (Fig. 1b)] from tadpoles showing clinical signs of chytridiomycosis (dekeratinized jaw sheath and tooth rows). We detected isolates from BdGPL-2 at all four farms and isolates from BdASIA-2/BdBRAZIL at two farms. One tadpole was infected with both lineages.

### Laboratory infection experiment

**Figure 4:**
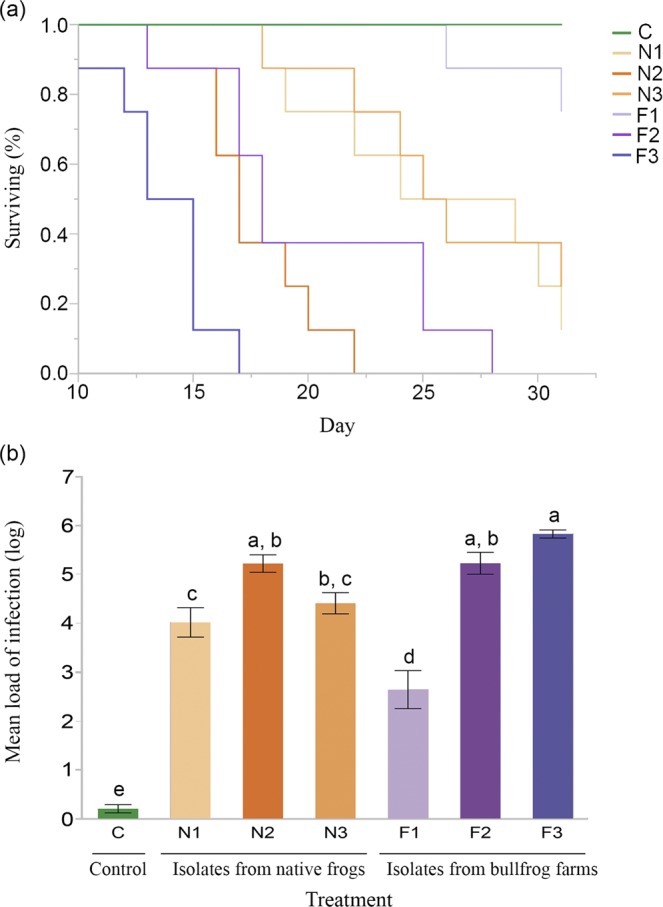
*Brachycephalus ephippium* survival curves (%) following inoculation with six Bd isolates (**a**); Mean infection load (log; ±SE) on day 16 (including of animals that died before day 16) among treatments (**b**).

We found a significant effect of Bd isolate on survival of the native toadlet *Brachycephalus ephippium* (χ^2^ = 37.269; df = 5; *P* < 0.0001; Fig. 4a). Isolates associated with the highest and lowest host mortality rates were both cultured from bullfrogs (Fig. 4a). However, Bd isolates cultured from bullfrogs and native frogs led to similar rates of host mortality (χ^2^ = 0.082; df = 1; *P* = 0.774).

Patterns in Bd infection loads were consistent with our survival analysis. Average pathogen loads differed among *B. ephippium* exposed to different Bd isolates (F_(6,49)_ = 68.918; *P* < 0.0001; Table 2, Fig. 4b, Supplementary Table S3). However, loads were similar between toadlets exposed to isolates cultured from bullfrogs and native frogs (F_(1,46)_ = 0.002; *P* = 0.965). Individuals that died during the experiment showed similarly high Bd infection loads (>10^5^ g.e.), independent of isolate (F_(5,35)_ = 1.674; *P* = 0.168; Table 3).

**Table 2:** Infection loads by Bd isolate during the experiment (day 16 and day of mortality); values presented as mean; SD (range; sample size). C = control; N = isolates from native amphibian hosts; F = isolates from bullfrogs.

Isolates	Day 16	Day of mortality
C	1; 1 (0–4; 8)	All survived
N1	31,261; 38,035 (330–102,645; 8)	828,748; 713,838 (166,029–1,786,135; 7)
N2	152,271; 130,841 (26,118–343,459; 5)	564,221; 214,328 (227,469–945,888; 8)
N3	47,925; 44,678 (2,147–120,497; 8)	693,070; 498,886 (104,653–1,465,878; 6)
F1	3,485; 5,863 (12–14,306; 8)	2,900,231; 1,208,707 (2,045,546–3,754,916; 2)
F2	260,637; 247,935 (14,698–561,934; 7)	788,856; 683,451 (250,975–2,147,927; 8)
F3	539,697 (1)	714,814; 420,074 (216,539–1,281,582; 8)

**Table 3:** Proportional Hazard analysis testing the interactive effects of Bd infection load and Bd isolates on the survival of *B. ephippium*.

Source	Nparm	Df	L-R χ^2^	Prob > ChiSq
Isolate	5	5	17.8006587	0.0032*
Loadlog	1	1	6.84387369	0.0089*
Isolate*loadlog	5	5	6.58048188	0.2538

## Discussion

Past studies have shown that invasive bullfrog populations harbor high prevalences of Bd and may contribute to the global spread of Bd through the international food trade^8,11,12^. However, we lack information on how frog farming practices may facilitate the spread of Bd to nearby native host communities and whether bullfrog farms function as a reservoir for highly virulent Bd strains. Our study demonstrates that bullfrog farms constantly release substantial quantities of Bd zoospores into the surrounding natural environment. The high concentration of Bd zoospores in frog pens probably results from movement of water among infected frog pens^36,37^. Releases of Bd zoospores from bullfrog farms may not only maintain the presence of Bd in the natural environment, but also may introduce the pathogen to new sites.

The high prevalence and infection loads observed in farmed bullfrogs can be explained by the high densities of frogs in these farms, which promotes pathogen transmission by direct contact among individuals^38^ or by circulating through frog pens^36,37^. Ideally, the density of animals should not surpass 20 tadpoles per liter of water, or 100 juveniles/50 adults per m^2^ ^33,39,40^. We observed high bullfrog densities of all frog life stages at sampled farms (Supplementary Fig. S1). Thus, stress caused by high host densities in captivity may lower host immune capacity and increase pathogen loads^39^.

We found lower Bd prevalence in bullfrog tadpoles than in juveniles and adults. Prevalence of Bd in farmed bullfrog tadpoles approximated or exceeded prevalences found in wild tadpoles in the Brazilian Atlantic Forest and savannah (Cerrado)^34^, the biomes where our sampled farms were located. Although visual inspection can be used as a proxy for Bd diagnosis^34,36,38,41,42^, it is possible that mouthpart dekeratinization may not be observed at early stages of infection^36^; thus, the prevalence we detected in bullfrog farms may be an underestimate. When we applied the error estimation to our raw data, Bd prevalence increased significantly, making our findings even worse. The above method uses different species and environmental conditions, and considers individuals Bd^+^ with zoospore genomic equivalents (g.e.) ≥0.1, while the present study ≥1 g.e. This may be generating an overestimated false negative value, however, considering the legitimacy of these data, the prevalence in bullfrog tadpoles may be higher than observed, and therefore, therefore we are most concerned about the high prevalence found in the bullfrog farms. Hence, we argue that tadpoles in bullfrog farms may play a key role as reservoirs of the pathogen^43^, maintaining high concentrations of zoospores in the water and promoting infection or reinfection of individuals.

We showed that juveniles had higher Bd prevalence compared to tadpoles, and higher infection loads compared to adults. Our findings corroborate other studies showing low infection loads in adults^43^ and juveniles with higher prevalence, infection loads, and mortality rates compared to adults^44^. During metamorphosis, tadpoles undergo several physiological processes that reshape the immune system, leading to stress that can increase susceptibility to Bd infection^45,46^. In addition, newly metamorphosed individuals may be more affected by Bd infection, since this is the stage at which keratinization of the skin occurs, providing new substrate and making them an excellent host for keratinophilic pathogens such as Bd^47^. In the wild, high prevalence and infection loads of juveniles may result in greater spread of Bd, since this stage of development is associated with high dispersion rates^48,49^.

The presence of Bd at all farms and host life stages suggests that this pathogen has the potential to interfere economically in ranaculture by negatively influencing the commercial production of bullfrogs. Although this species tolerates Bd infection^23,26^, previous study shows that bullfrog tadpoles displayed multiple cardiac alterations in response to infection^50^, possibly incurring a high energy cost to developing animals, affecting metamorphosis and potentially reducing growth and post-metamorphic survival. Furthermore, other sublethal effects of Bd have been reported in tadpoles, such as behavioral changes and reduced feeding^51^. Chytrid infection may also reduce frog immune responses, increasing susceptibility to other infections^52^. Therefore, controlling chytrid infections in frog farms would potentially increase profits, due to enhanced growth and quality of frogs produced.

In addition to serving as a reservoir of the pathogen, bullfrogs may host high genetic diversity of Bd^53^. The presence of two Bd lineages within our focal farms, BdGPL and BdASIA-2/BdBRAZIL, even in the same tadpole, creates a high potential for hybridization. Hybrid lineages, such as the one between BdGPL × BdASIA-2/BdBRAZIL in Brazil’s Atlantic forest^28,54^, may be more virulent than the parental lineages^32^, in accordance with the hybrid vigor theory^55^. Hybridization of Bd lineages is apparently rare in the wild^11,54^, but frog farms may exacerbate the risk of new hybrids^32,56^.

The evolution of virulence is expected in bullfrog farms due to short generation times of Bd^57^, high host tolerance^23,26^, and high host population densities^38^. However, we found no support for higher virulence of isolates originating from bullfrog farms compared to those originating from wild frogs. Instead, individuals of *B. ephippium* died when Bd infection load surpassed 100,000 zoospores g.e., independent of isolate or origin (bullfrog or native host)^58^. In contrast, an infection load threshold of 10,000 zoospores was considered to be fatal for aquatic-breeding species^59,60^, indicating that these thresholds vary among species with contrasting life histories. The high variation in Bd load that we observed between host individuals could be attributed to multiple factors that can act alone or synergistically. Variation in innate and adaptive host immune responses^61,62^, gene expression^63,64^, previous contact with the pathogen^65^ and host stress levels^66^ may modulate the intensity of infection. Also, infections from different strains may result in different Bd loads^32^.

Our study emphasizes the prominent role that bullfrog farms may play in the spread of Bd into native host communities. In addition, it suggests that Bd strains that evolve on bullfrog farms are likely to impact susceptible native species through the release of zoospores into the environment, a form of pathogen spread. Our results indicate that bullfrog farms, which are distributed across the world, provide a potential environment for survival, reproduction and dissemination of Bd inside and outside farms. A possible way to reduce the spread of chytrid fungus through farming would be to treat the water that is released to the environment. Bullfrogs should also be treated for Bd infection using previously reported treatment methods^67^. Additional studies aimed at treatment and control of Bd in amphibian farming systems would be beneficial to native amphibian conservation efforts.

## Materials and Methods

### Bullfrog farm assessment

We sampled 10 bullfrog farms in the state of São Paulo, located in Brazilian Atlantic Forest and savannah (Cerrado), southeastern Brazil. Our sampling quantified Bd prevalence and infection loads in farmed bullfrogs. We sampled 35 juveniles from each of eight farms and 35 adults from each of nine farms. For each individual, we swabbed the inguinal region and each limb 5 times, specifically between the digits^68^. We used disposable gloves to handle each individual frog to avoid cross-contamination. Additionally, we sampled 100 tadpoles from each of nine farms for the presence of Bd (Supplementary Fig. S2a). We selected tadpoles at Gosner’s stage 25 and visually inspected jaws sheaths and tooth rows. We did not use molecular methods of detection for tadpoles because dekeratinization of the oral region is a reliable proxy for Bd infection in tadpoles^36,41^, including amphibians sampled in Brazil^34,69^. We considered individuals with jaw sheath dekeratinization to be infected with Bd (Bd^+^), regardless of the level of dekeratinization of the tooth rows (Supplementary Fig. S2). Although visual inspection for mouthpart dekeratinization is used for Bd diagnosis in tadpoles^34,41^, some studies show that tadpoles with early stages of infection may not present mouthpart dekeratinization^36^, and that tadpoles with fully dekeratinized mouthparts no longer have substrate for Bd growth^34^, therefore leading to false positives and negatives^42^. Thus, in order to validate our method, we ran qPCR analyses with a subsample of 51 bullfrog tadpoles with dekeratinized mouthparts from one of the focal bullfrog farms. We found a very low proportion of false positives (two individuals = 4%), indicating that visual inspection for mouthpart dekeratinization is a realiable method for Bd detection in bullfrogs from the state of São Paulo (Supplementary Table S4). In addition, and to be even more conservative, we included an estimation of false negatives and false positives in our analyses (see below) based on previous studies. We applied as a possible error the largest estimates observed in a recent study^42^: 12.3% for false positives and 18.7% for false negatives (Supplementary Table S1).

In addition to detecting and quantifying Bd from individual frogs, we collected water samples and standardized a filtering protocol to detect the pathogen in the aquatic environment. We measured the volume of water released by farms into the surrounding natural environment (L/s). We measured outflow rate as the amount of water released per second. Then we collected 1 liter and filtered 500 ml of this water from each farm (Supplementary Fig. S1d). Using a vacuum pump, we filtered the sampled water with a permeable membrane (0.45 μm pore size) (Supplementary Fig. S3). After filtering, we extracted DNA from membranes and quantified Bd zoospores and zoosporangia using qPCR as described below.

#### qPCR, Bd isolation and sequencing

We extracted Bd DNA from swab samples of juveniles and adult amphibians using PrepMan ULTRA (Life Technologies) and performed quantitative PCR analyses for Bd detection and quantification^68^. We considered samples with zoospore genomic equivalents (g.e.) ≥1 to be Bd^+^ ^70^. We estimated Bd infection prevalence (for tadpoles, juveniles and adults) as the number of infected individuals divided by the total number of sampled individuals for each farm.

We cultured Bd from bullfrog tadpoles showing mouthpart dekeratinization, following protocols by Vieira & Toledo^71^ and Fisher *et al*.^72^. After isolation, we transferred Bd cultures into Petri dishes containing 1% Tryptone agar and incubated cultures for one week. We then extracted DNA from the culture, following protocols by James *et al*.^73^. We genotyped each Bd isolate using a sequence of 6 SNP markers (Supplementary Table S5) as described by Schloegel *et al*.^28^ and sequenced them in the Sequencing Core Lab at the University of Michigan.

### Laboratory infection experiment

We conducted experimental inoculations in the laboratory to test for effects of different Bd isolates on amphibian hosts. We used *Brachycephalus ephippium* (Anura: Brachycephalidae) as an experimental host. *Brachycephalus ephippium* is a direct-developing species endemic to Brazil’s Atlantic Forest^74^. Direct developers carry low Bd prevalence in the wild and often show low resistance to chytridiomycosis^58^. Thus, they are an ideal model organism for infection trials. We collected *B. ephippium* (about 2 cm in snout-vent length) in the municipality of Mogi das Cruzes, state of São Paulo, Brazil. We individually housed each wild-caught individual in plastic bags with leaf-litter to avoid potential cross-contamination among frogs while in the field. We swabbed all individual frogs and only used those that tested negative for Bd in the field. During the experiment, we individually housed each frog in plastic boxes (22 × 15 × 8 cm) containing autoclaved moist *Sphagnum* moss. We monitored frogs daily and fed them calcium-fortified pinhead crickets. We carried out the experiment in a temperature-controlled room, with temperatures set at 20 °C and a 12 h day-night cycle.

We exposed frogs to three Bd isolates from bullfrogs sampled at farms for this study and three Bd isolates previously isolated and genotyped from native amphibian hosts. Our experimental design consisted of seven treatments (six Bd isolates and a negative control) with eight frogs per treatment (Supplementary Table S6). All isolates were within the BdGPL-2 clade^28^, which is the dominant form in the Brazilian Atlantic Forest^54^. We cultured Bd isolates in Petri dishes with tryptone agar at 17 °C for five days. We then harvested Bd zoospores by flooding Petri dishes with distilled water and waiting for approximately one hour for zoospore release from zoosporangia^32,75^. We then quantified zoospores in a Neubauer hemocytometer and standardized the inoculum concentration (4.6 × 10^6^ zoospores/ml) among isolates.

For inoculations, we placed frogs in individual Petri dishes containing 1 ml of Bd inoculum (treatment) or 1 ml of autoclaved distilled water (control) for 45 minutes. This procedure occurred only once for each animal. We swabbed each individual 16 and 31 days following inoculation, which is sufficient time for multiple Bd generations^76^. We monitored amphibians daily and swabbed dead or dying individuals. Experimental protocols were approved by the local animal care committee (CEUA UNICAMP #4688-1/2017). We used the same DNA extraction and qPCR protocols described above to detect and quantify Bd^68^.

### Statistical analyses

#### Bullfrog farm assessment

We used a General Linear Model (GLM) with a binomial distribution (logit link) to test for differences in Bd prevalence among host developmental stages (tadpole, juvenile and adult); we performed a Tukey HSD *a posteriori* test for multiple comparisons. In addition, we repeated this analysis taking into account the estimated rates of false negatives and false positives associated with the visual inspection method. We also ran a GLM with normal distribution (identity link) to test for differences in Bd infection loads among host developmental stage (juvenile and adult). We log-transformed (log10) infection load data to obtain GLMs with normally distributed residuals.

#### Laboratory infection experiment

We built survival curves (Parametric Survival analyses) to test for effects of isolation source (native frogs vs. bullfrogs) on the survival of individual hosts. We performed a similar analysis to test for effects of isolate on host survival. In addition, we used a Generalized Linear Mixed Model (GLMM) to test whether Bd infection loads in *B. epphipium* exposed to bullfrog Bd isolates were higher than those exposed to isolates from native frogs, including the six Bd isolates as a random effect. For these analyses, we included samples collected halfway through the experiment (day 16) and included swabs of individuals that died before day 16; we excluded the control group from this analysis. We also performed a Tukey HSD *a posteriori test* for pairwise multiple comparisons among means. We also used a simple Analysis of Variance (ANOVA) to test for differences in average infection loads at the point of mortality among individuals exposed to different isolates. Finally, we used Proportional Hazards analysis, including the interaction between infection load and isolates, to test whether survival depended on these factors. We also excluded the control group from this analysis.

### Ethics statement

All experiments were performed in accordance with university guidelines and regulations for animal care and husbandry. Our collecting permit was provided by ICMBio (SISBio #54656-3; 27745-13; 17242-3). Experimental protocols were approved by Universidade Estadual de Campinas (UNICAMP) and the local animal care committee [Comissão de Ética no Uso de Animal – CEUA (#4688-1/2017)]. This research was accessioned at SISGen platform (SISGEN #A1E9E10).

## Supplementary information

Supplementary information

## Data Availability

The datasets generated during and/or analyzed during the current study are available from the corresponding author on reasonable request.

## References

[1] Barrasso DA, Cajade R, Nenda SJ, Baloriani G, Herrera R (2009). Introduction of the American bullfrog *Lithobates catesbeianus* (Anura: Ranidae) in natural and modified environments: an increasing conservation problem in Argentina. S. Am. J. Herpetol..

[2] Frost, D. R. Amphibian Species of the World: an Online Reference. Version 6.0. American Museum of Natural History, New York, USA. Available from http://research.amnh.org/herpetology/amphibia/index.html (accessed March 2018) (2018).

[3] FAO (Food and Agriculture Organization) 2005–2018. Cultured Aquatic Species Information Programme - *Rana catesbeiana*. Text by Flores Nava, A. In FAO Fisheries and Aquaculture Department, Rome, http://www.fao.org/fishery/culturedspecies/Rana_catesbeiana/en (2018).

[4] Carpenter, A. I. *et al*. Over-harvesting. Pages 26–31 in Gascon, C. *et al*. editors. Amphibian conservation action plan. IUCN/SSC Amphibian Specialist Group, Gland, Switzerland and Cambridge, UK (2007).

[5] Kats LB, Ferrer RP (2003). Alien predators and amphibian declines: review of two decades of science and the transition to conservation. Divers. Distrib..

[6] Fisher MC, Garner TW (2007). The relationship between the emergence of *Batrachochytrium dendrobatidis*, the international trade in amphibians and introduced amphibian species. Fungal Biol. Rev..

[7] Laufer G, Canavero A, Núñez D, Maneyro R (2008). Bullfrog (*Lithobates catesbeianus*) invasion in Uruguay. Biol. Invasions.

[8] Schloegel LM (2009). Magnitude of the US trade in amphibians and presence of *Batrachochytrium dendrobatidis* and ranavirus infection in imported North American bullfrogs (*Rana catesbeiana*). Biol. Conserv..

[9] Carpenter AI, Andreone F, Moore RD, Griffiths RA (2014). A review of the international trade in amphibians: the types, levels and dynamics of trade in CITES-listed species. Oryx.

[10] GISD (Global Invasive Species Database). Available from, http://193.206.192.138/gisd/search.php (accessed March 2018) (2018).

[11] O’Hanlon SJ (2018). Recent Asian origin of chytrid fungi causing global amphibian declines. Science.

[12] Garner TW (2006). The emerging amphibian pathogen *Batrachochytrium dendrobatidis* globally infects introduced populations of the North American bullfrog, *Rana catesbeiana*. Biol. Letters.

[13] Lau, M., van Dijk, P. P. & Syed, G. P. Managing problems of overexploitation and trade in amphibians. In Stuart, S. *et al*. editors. *Threatened Amphibians of the World, Lynx Edicions, Barcelona* (2008).

[14] Both C (2011). Widespread occurrence of the American bullfrog, *Lithobates catesbeianus* (Shaw, 1802) (Anura: Ranidae), in Brazil. S. Am. J. Herpetol..

[15] Medeiros CI, Both C, Grant T, Hartz SM (2017). Invasion of the acoustic niche: variable responses by native species to invasive American bullfrog calls. Biol. Invasions.

[16] Forti LR (2017). Perspectives on invasive amphibians in Brazil. PLoS One.

[17] Toledo LF, Silva RR, Haddad CFB (2007). Anurans as prey: an exploratory analysis and size relationships between predators and their prey. J. Zool..

[18] Leivas PT, Savaris M, Lampert S, Lucas EM (2013). Predation of *Odontophrynus americanus* (Anura: Odontophrynidae) by the invasive species *Lithobates catesbeianus* (Anura: Ranidae) in an Araucaria Forest remnant in Southern Brazil. Herpetol. Notes.

[19] Kiesecker JM, Blaustein AR, Miller CL (2001). Potential mechanisms underlying the displacement of native red‐legged frogs by introduced bullfrogs. Ecology.

[20] Boone MD, Little EE, Semlitsch RD (2004). Overwintered bullfrog tadpoles negatively affect salamanders and anurans in native amphibian communities. Copeia.

[21] Scheele BC (2019). Amphibian fungal panzootic causes catastrophic and ongoing loss of biodiversity. Science.

[22] Hanselmann R (2004). Presence of an emerging pathogen of amphibians in introduced bullfrogs *Rana catesbeiana* in Venezuela. Biol. Conserv..

[23] Eskew EA, Worth SJ, Foley JE, Todd BD (2015). American bullfrogs (*Lithobates catesbeianus*) resist infection by multiple isolates of *Batrachochytrium dendrobatidis*, including one implicated in wild mass mortality. EcoHealth.

[24] Mazzoni R (2003). Emerging pathogen in wild amphibians and frogs (*Rana catesbeiana*) farmed for international trade. Emerg. Infect. Dis..

[25] Gervasi SS (2013). Experimental evidence for American Bullfrog (*Lithobates catesbeianus*) susceptibility to chytrid fungus (*Batrachochytrium dendrobatidis*). EcoHealth.

[26] Daszak P (2004). Experimental evidence that the bullfrog (*Rana catesbeiana*) is a potential carrier of chytridiomycosis, an emerging fungal disease of amphibians. Herpetol. J..

[27] Kriger KM, Hero J (2009). Chytridiomycosis, Amphibian Extinctions, and Lessons for the Prevention of Future Panzootics. EcoHealth.

[28] Schloegel LM (2012). Novel, panzootic and hybrid genotypes of amphibian chytridiomycosis associated with the bullfrog trade. Mol. Ecol..

[29] Farrer RA (2011). Multiple emergences of genetically diverse amphibian-infecting chytrids include a globalized hypervirulent recombinant lineage. P. Natl. Acad. Sci. USA.

[30] Rosenblum EB (2013). Complex history of the amphibian-killing chytrid fungus revealed with genome resequencing data. P. Natl. Acad. Sci. USA.

[31] Becker CG (2017). Variation in phenotype and virulence among enzootic and panzootic amphibian chytrid lineages. Fungal Ecol..

[32] Greenspan SE (2018). Hybrids of amphibian chytrid show high virulence in native hosts. Sci. Rep..

[33] Ferreira CM, Pimenta AGC, Paiva-Neto JS (2002). Introdução à ranicultura. Bol. Inst. Pesca.

[34] Carvalho T, Becker CG, Toledo LF (2017). Historical amphibian declines and extinctions in Brazil linked to chytridiomycosis. P. R. Soc. B..

[35] Brito MFG (2018). Brazil naturalizes non-native species. Science.

[36] Rachowicz LJ, Vredenburg VT (2004). Transmission of *Batrachochytrium dendrobatidis* within and between amphibian life stages. Dis. Aquat. Organ..

[37] Berger L, Hyatt AD, Speare R, Longcore JE (2005). Life cycle stages of the amphibian chytrid *Batrachochytrium dendrobatidis*. Dis. Aquat. Organ..

[38] Rachowicz LJ, Briggs CJ (2007). Quantifying the disease transmission function: effects of density on *Batrachochytrium dendrobatidis* transmission in the mountain yellow‐legged frog *Rana muscosa*. J. Anim. Ecol..

[39] Cribb, A. Y., Afonso, A. M. & Mostério, C. M. F. Manual Técnico de Ranicultura. Embrapa Agroindústria de Alimentos, Brasília, 13–71 (2013).

[40] Seixas Filho, J. T., Pereira, M. M. & Mello, S. C. R. P. Manual de Ranicultura para o Ranicultor. H. P. Comunicação, **155** (2017).

[41] Knapp RA, Morgan JA (2006). Tadpole mouthpart depigmentation as an accurate indicator of chytridiomycosis, an emerging disease of amphibians. Copeia.

[42] Navarro-Lozano A, Sánchez-Domene D, Rossa-Feres DC, Bosch J, Sawaya RJ (2018). Are oral deformities in tadpoles accurate indicators of anuran chytridiomycosis?. PloS One.

[43] Briggs CJ, Knapp RA, Vredenburg VT (2010). Enzootic and epizootic dynamics of the chytrid fungal pathogen of amphibians. P. Natl Acad Sci USA B.

[44] Langhammer PF, Burrowes PA, Lips KR, Bryant AB, Collins JP (2014). Susceptibility to the amphibian chytrid fungus varies with ontogeny in the direct-developing frog, *Eleutherodactylus coqui*. J. Wildlife Dis..

[45] Rollins‐Smith LA (1998). Metamorphosis and the amphibian immune system. Immunol. Rev..

[46] Fernández-Loras A, Fernández-Beaskoetxea S, Arriero E, Fisher MC, Bosch J (2017). Early exposure to *Batrachochytrium dendrobatidis* causes profound immunosuppression in amphibians. Eur. J. Wildlife Res..

[47] Berger L (1998). Cytridiomycosis causes amphibian mortality associated with population declines in the rain forests of Australia and Central America. P. Natl. Acad. Sci USA.

[48] Hamilton WD, May RM (1977). Dispersal in stable habitats. Nature.

[49] Semlitsch RD (2008). Differentiating migration and dispersal processes for pond‐breeding amphibians. J. Wildlife Manage..

[50] Salla RF (2015). Cardiac adaptations of bullfrog tadpoles in response to chytrid infection. J. Exp. Zool..

[51] DeMarchi JA, Gaston JR, Spadaro AN, Porterfield CA, Venesky MD (2015). Tadpole food consumption decreases with increasing *Batrachochytrium dendrobatidis* infection intensity. J. Herpetol..

[52] Miller DL (2008). Concurrent infection with ranavirus, *Batrachochytrium dendrobatidis*, and *Aeromonas* in a captive anuran colony. J. Zoo Wildlife Med..

[53] Goka K (2009). Amphibian chytridiomycosis in Japan: distribution, haplotypes and possible route of entry into Japan. Mol. Ecol..

[54] Jenkinson TS (2016). Amphibian-killing chytrid in Brazil comprises both locally endemic and globally expanding populations. Mol. Ecol..

[55] Whaley WG (1944). Heterosis. Bot. Rev..

[56] Ghosh P, Fisher MC (2016). Dr Jekyll and Mrs Hyde: Risky hybrid sex by amphibian-parasitizing chytrids in the Brazilian Atlantic Forests. Mol. Ecol..

[57] Hamilton WD (1980). Sex versus non-sex versus parasite. Oikos.

[58] Mesquita AF (2017). Low resistance to chytridiomycosis in direct-developing amphibians. Sci. Rep..

[59] Vredenburg VT, Knapp RA, Tunstall TS, Briggs CJ (2010). Dynamics of an emerging disease drive large-scale amphibian population extinctions. P. Natl. Acad. Sci. USA.

[60] Kinney VC, Heemeyer JL, Pessier AP, Lannoo MJ (2011). Seasonal pattern of *Batrachochytrium dendrobatidis* infection and mortality in *Lithobates areolatus*: Affirmation of Vredenburg’s “10,000 Zoospore Rule”. Plos One.

[61] Ramsey JP, Reinert LK, Harper LK, Woodhams DC, Rollins-Smith LA (2010). Immune defenses against *Batrachochytrium dendrobatidis*, a fungus linked to global amphibian declines, in the South African clawed frog, *Xenopus laevis*. Infect. Immun..

[62] Voyles J, Rosenblum EB, Berger L (2011). Interactions between *Batrachochytrium dendrobatidis* and its amphibian hosts: a review of pathogenesis and immunity. Microbes Infect..

[63] Savage AE, Zamudio KR (2011). MHC genotypes associate with resistance to a frog-killing fungus. P. Natl. Acad. Sci. USA.

[64] Bataille A (2015). Susceptibility of amphibians to chytridiomycosis is associated with MHC class II conformation. Proc. Biol. Sci..

[65] McMahon TA (2014). Amphibians acquire resistance to live and dead fungus overcoming fungal immunosuppression. Nature.

[66] Gabor CR, Fisher MC, Bosch J (2013). A non-invasive stress assay shows that tadpole populations infected with *Batrachochytrium dendrobatidis* have elevated corticosterone levels. PloS One.

[67] Moreno LF, Morão P, Toledo LF (2015). Tratamento de anfíbios infectados pelo fungo quitrídio do gênero Batrachochytrium. Herpetol. Bras..

[68] Lambertini C, Rodriguez D, Brito FB, Leite DS, Toledo LF (2013). Diagnóstico do fungo Quitrídio: *Batrachochytrium dendrobatidis*. Herpetol. Bras..

[69] Vieira CA, Toledo LF, Longcore JE, Longcore JR (2013). Body length of *Hylodes cf. ornatus* and *Lithobates catesbeianus* tadpoles, depigmentation of mouthparts, and presence of *Batrachochytrium dendrobatidis* are related. Braz. J. Biol..

[70] Kriger KM, Hiner HB, Hyatt AD, Boyle DG, Hero JM (2006). Techniques for detecting chytridiomycosis in wild frogs: comparing histology with real-time Taqman PCR. Dis. Aquat. Organ..

[71] Vieira CA, Toledo LF (2012). Isolamento, cultivo e armazenamento do fungo quitrídio: *Batrachochytrium dendrobatidis*. Herpetol. Bras..

[72] Fisher MC (2018). Development and worldwide use of non-lethal, and minimal population-level impact, protocols for the isolation of amphibian chytrid fungi. Sci. Rep..

[73] James TY, Stenlid J, Olson A, Johannesson H (2008). Evolutionary significance of imbalanced nuclear ratios within heterokaryons of the basidiomycete fungus *Heterobasidion parviporum*. Evolution.

[74] Haddad, C. F. *et al*. Guia dos anfíbios da Mata Atlântica: diversidade e biologia. Anolis Books, **542** (2013).

[75] Jenkinson TS (2018). Globally invasive genotypes of the amphibian chytrid outcompete an enzootic lineage in coinfections. P. R. Soc. B.

[76] Longcore JE, Pessier AP, Nichols DK (1999). *Batrachochytrium dendrobatidis* gen. et sp. nov., a chytrid pathogenic to amphibians. Mycologia.

