# Supplementary material for: Bullfrog farms release virulent zoospores of the frog-killing fungus into the natural environment
Source: Sci Rep. 2019 Sep 17;9:13422. doi: 10.1038/s41598-019-49674-0 (PMC6748994; doi:10.1038/s41598-019-49674-0)
Supplement: Supplementary file 1 — Supplementary information [file 41598_2019_49674_MOESM1_ESM.pdf]

Bullfrog farms release virulent zoospores of the frog-killing fungus into the natural environment

Luisa P. Ribeiro[1*], Tamilie Carvalho[1], C. Guilherme Becker[2], Thomas S. Jenkinson[3], Domingos da Silva Leite[4], Timothy Y. James[3], Sasha E. Greenspan[2], Luís Felipe Toledo[1]

[1] Laboratório de História Natural de Anfíbios Brasileiros (LaHNAB), Instituto de Biologia, Universidade Estadual de Campinas (UNICAMP), CEP 13083-862, Campinas, São Paulo, Brasil

[2] Department of Biological Sciences, The University of Alabama, Tuscaloosa, Alabama 35487, USA

[3] Department of Ecology and Evolutionary Biology, University of Michigan, Ann Arbor, Michigan, 48109, USA

[4] Departamento de Genética, Evolução, Microbiologia e Imunologia, Instituto de Biologia, Universidade Estadual de Campinas (UNICAMP), CEP 13083-862, Campinas, São Paulo, Brasil

*Corresponding author: Luisa P. Ribeiro, Universidade Estadual de Campinas,

lupribeiro70@gmail.com

Supplementary Information

Supplementary Table S1. Proportion of false negatives [tadpoles incorrectly classified as Bd^+/total individuals tested (percentage)] and false positives [tadpoles incorrectly classified as Bd^-/total individuals tested (percentage)] based on a comparison of the visual inspection method with different verification methods from previous studies and the present study (bullfrog tadpoles from a frog farm in the state of São Paulo, Brazil).

Reference	Method	False negatives	False positives
Carvalho et al. 2017	qPCR	2/14 (14.3 %)	0/10 (0 %)
Carvalho et al. 2017	Histology	1/10 (10 %)	0/10 (0 %)
Carvalho et al. 2017	Bd isolation success	6/54 (11 %)	0/120 (0 %)
Navarro-Lozano et al. 2018	qPCR	45/241 (18.7 %)	17/183 (12.3 %)
Present study	qPCR	-	2/51 (4 %)
Present study	Bd isolation success	-	0/6 (0 %)

Supplementary Table S2. Results of general linear models comparing Bd prevalence among three developmental stages (tadpole, juvenile and adult). Observed data: data obtained in the present study; False positive and false negative: data obtained in the present study accounting for the highest percentage of false positives (less 12.3%) and false negatives (more 18.7%), according to Navarro-Lozano et al. (2018). J = juvenile, A = adult, T = tadpole. Bold values are significant.

	**F**	***P***	**Tukey *P***
Observed data without error included	4.226	**0.027**	J-A 0.7<br>T-A 0.1<br>**T-J 0.02***
False positive error included	4.767	**0.018**	J-A 0.7<br>T-A 0.08<br>**T-J 0.01***
False negative error included	1.698	0.2	J-A 0.7<br>T-A 0.5<br>T-J 0.1

Supplementary Table S3. Bd infection load in *B. ephippium* on days 16, 31, and on the day animals died along the experiment. The day the individual died is stated in parenthesis.

Individual	Treatment	Genotype	Infection load (zoospore g.e.)		
			**Day 16**	**Day 31**	**Day of mortality**
1	Control	C	0	Not analyzed	Survived
2	Control	C	0	Not analyzed	Survived
3	Control	C	1	Not analyzed	Survived
4	Control	C	0	Not analyzed	Survived
5	Control	C	3	Not analyzed	Survived
6	Control	C	0	Not analyzed	Survived
7	Control	C	0	Not analyzed	Survived
8	Control	C	0	Not analyzed	Survived
9	Nature	N1	330	9,756	Survived
10	Nature	N1	1,809	Dead	1,786,135 (30)
11	Nature	N1	102,645	Dead	166,029 (18)
12	Nature	N1	8,964	Dead	444,168 (24)
13	Nature	N1	4,329	Dead	1,632,237 (31)
14	Nature	N1	32,826	Dead	1,289,281 (29)
15	Nature	N1	23,999	Dead	299,811 (22)
16	Nature	N1	75,184	Dead	183,578 (19)
17	Nature	N2	26,118	Dead	462,761 (22)
18	Nature	N2	164,616	Dead	657,068 (19)
19	Nature	N2	Dead	Dead	414,355 (16)
20	Nature	N2	Dead	Dead	653,676 (13)
21	Nature	N2	343,459	Dead	656,325 (17)
22	Nature	N2	Dead	Dead	227,470 (16)
23	Nature	N2	33,383	Dead	496,228 (20)
24	Nature	N2	193,779	Dead	945,888 (17)
25	Nature	N3	2,147	Dead	691,309 (31)
26	Nature	N3	8,213	76,299	Survived
27	Nature	N3	9,435	55,530	Survived
28	Nature	N3	102,780	Dead	358,793 (22)
29	Nature	N3	38,641	Dead	1,066,992 (24)
30	Nature	N3	65,673	Dead	470,795 (25)
31	Nature	N3	36,013	Dead	1,465,878 (26)
32	Nature	N3	120,497	Dead	104,653 (18)
33	Farm	F1	77	20	Survived
34	Farm	F1	325	195,952	Survived
35	Farm	F1	12	0	Survived
36	Farm	F1	14,306	Dead	2,045,546 (31)
37	Farm	F1	11,472	Dead	3,754,916 (26)
38	Farm	F1	38	8,623	Survived
39	Farm	F1	766	14,937	Survived
40	Farm	F1	884	17,904	Survived
41	Farm	F2	Dead	Dead	773,820 (13)

Individual	Treatment	Genotype	Infection load (zoospore g.e.)		
			Day 16	Day 31	Day of mortality
42	Farm	F2	547,360	Dead	396,831 (17)
43	Farm	F2	561,934	Dead	266,903 (17)
44	Farm	F2	14,699	Dead	2,147,927 (28)
45	Farm	F2	39,210	Dead	1,486,504 (25)
46	Farm	F2	91,081	Dead	250,975 (25)
47	Farm	F2	452,991	Dead	646,158 (18)
48	Farm	F2	117,189	Dead	341,727 (18)
49	Farm	F3	539,697	Dead	216,539 (17)
50	Farm	F3	Dead	Dead	735,149 (10)
51	Farm	F3	Dead	Dead	303,891 (13)
52	Farm	F3	Dead	Dead	388,178 (12)
53	Farm	F3	Dead	Dead	523,255 (15)
54	Farm	F3	Dead	Dead	1,131,023 (13)
55	Farm	F3	Dead	Dead	1,281,582 (15)
56	Farm	F3	Dead	Dead	1,138,894 (15)

Supplementary Table S4. qPCR results (positive or negative) and Bd infection load for farmed bullfrog tadpoles identified as Bd-positive by the visual inspection method, based on mouthpart dekeratinization (as in Figure S2). In bold the two false positives (accordingly to the qPCR) we misdiagnosed.

Tadpole ID	Result	Load (zoospore g.e.)
GRSP 07	Positive	69.2
GRSP 08	Positive	188.4
GRSP 09	Positive	185.7
GRSP 10	Positive	397.7
GRSP 11	Positive	391.9
**GRSP 12**	**Negative**	**0**
GRSP 13	Positive	88.5
GRSP 14	Positive	75.6
GRSP 15	Positive	233.5
GRSP 16	Positive	4201.7
GRSP 17	Positive	57.4
GRSP 18	Positive	386.5
GRSP 19	Positive	20.9
GRSP 20	Positive	78.4
GRSP 21	Positive	118.6
GRSP 22	Positive	158.1
GRSP 24	Positive	17.9
GRSP 25	Positive	16.1
GRSP 26	Positive	88.4
GRSP 27	Positive	6.4
GRSP 28	Positive	24.3
GRSP 29	Positive	180.4
GRSP 30	Positive	318.1
GRSP 31	Positive	119.5
GRSP 32	Positive	163.1
GRSP 33	Positive	96
GRSP 34	Positive	55.3
GRSP 35	Positive	489.3
GRSP 36	Positive	643.5
GRSP 37	Positive	8.1
GRSP 38	Positive	12
GRSP 39	Positive	40.4
GRSP 40	Positive	29.1
GRSP 41	Positive	122.7
GRSP 42	Positive	12.4
GRSP 43	Positive	343.4
GRSP 44	Positive	13.5
GRSP 45	Positive	123.5
GRSP 46	Positive	22.8
GRSP 47	Positive	60.5
GRSP 48	Positive	220.6

Tadpole ID	Result	Load (zoospore g.e.)
GRSP 49	Positive	90.5
GRSP 50	Positive	17
GRSP 51	Positive	177.2
GRSP 52	Positive	19
**GRSP 53**	**Negative**	**0**
GRSP 54	Positive	47.9
GRSP 55	Positive	320.4
GRSP 56	Positive	204.5
GRSP 57	Positive	152.4
GRSP 58	Positive	285

Supplementary Table S5. Multilocus sequence typing (MLST) markers genotyped for this study.

Locus	PCR Primers	Anneal Temp.	Source
8009X2	F: 5’-TCGTGAAGAGCTTGGAAAGTCG-3’	54 °	Morgan *et al*. 2007
	R: 5’-AGTTCTGTCGTCAATGCTGTAGGG-3’		
BdC24	F: 5’-GACAATGTGCTCACGGCTTA-3’	54 °	James *et al*. 2009
	R: 5’-CTCTCCAAGGCTGAATCTGG-3’		
BdSC4.16	F: 5’-TCAACTGGCTTTGAGCACAC-3’	54 °	Schloegel *et al*. 2012
	R: 5’-ATAGAGCATGCAGATCGCTTT-3’		
R6046	F: 5’-CTATCTGCGCTCCCGTGTCAA-3’	54 °	Morehouse *et al*. 2003
	R: 5’-AGGGCTGCAACAACTGGATTT-3’		
BdSC6.15	F: 5’-GACGATAAAACGACAACAATCG-3’	54 °	Schloegel *et al*. 2012
	R: 5’-CCCTTTTTAGGTTGGCTTGC-3’		
BdSC8.10	F: 5’-TGACAAAGTGCCGAGTGTTT-3’	54 °	Schloegel *et al*. 2012
	R: 5’-TTGGCTATACCCGACTACGC-3’		

Supplementary Table S6. *Batrachochytrium dendrobatidis* (Bd) genotypes used in experimental inoculations. Hosts species, locality (municipality, state), year of isolation, and approximate number of passages are given for each genotype isolated.

Strains	Host species	Locality	Year	Passages
**N1**	*Bokermannohyla* sp.	Santa Teresa, ES	2014	8
**N2**	*Megaelosia apuana*	Alto Caparaó, MG	2015	5
**N3**	*Ololygon hiemalis*	Jundiaí, SP	2011	12
**F1**	*Lithobates catesbeianus*	Pindamonhangaba, SP	2016	3
**F2**	*Lithobates catesbeianus*	Santa Bárbara D'Oeste, SP	2016	3
**F3**	*Lithobates catesbeianus*	Santa Isabel, SP	2016	3

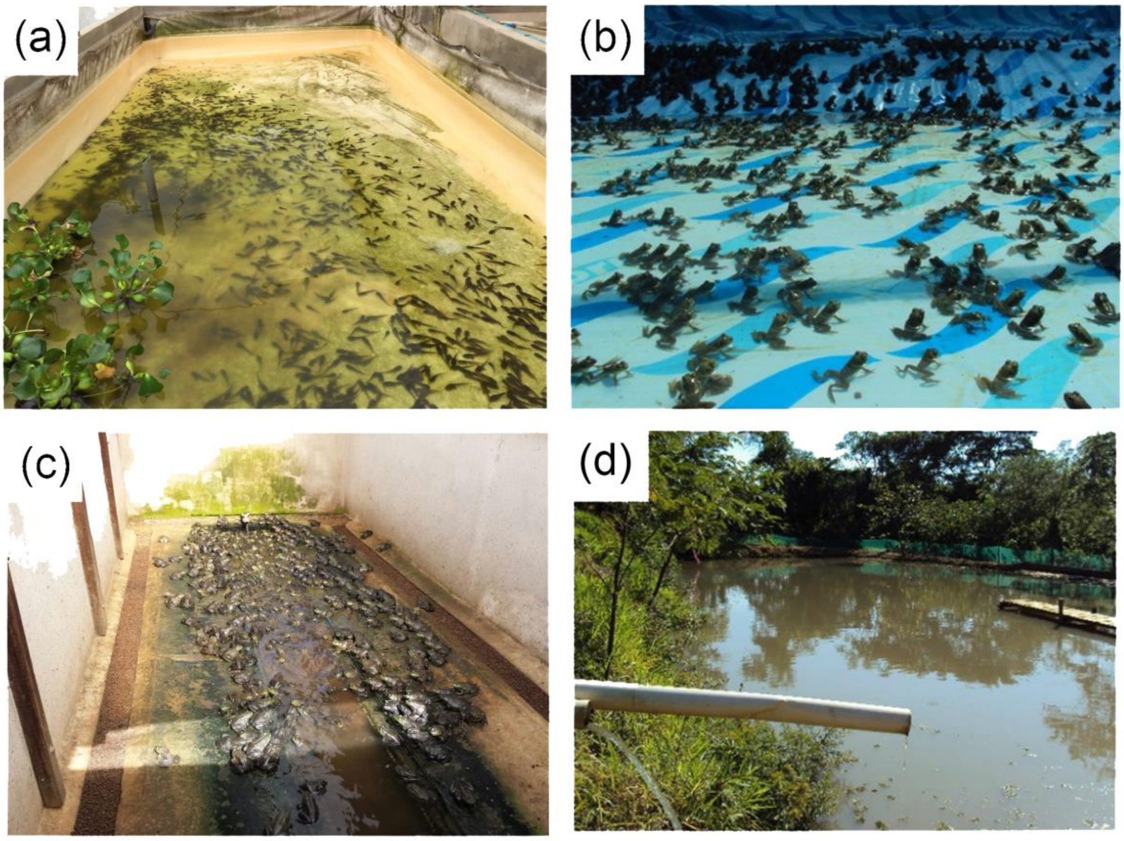

Supplementary Figure S1. Bullfrog farming system. Tadpoles (a), juveniles (b), and adults (c) sectors; and water released by the farms into an adjacent pond (d).

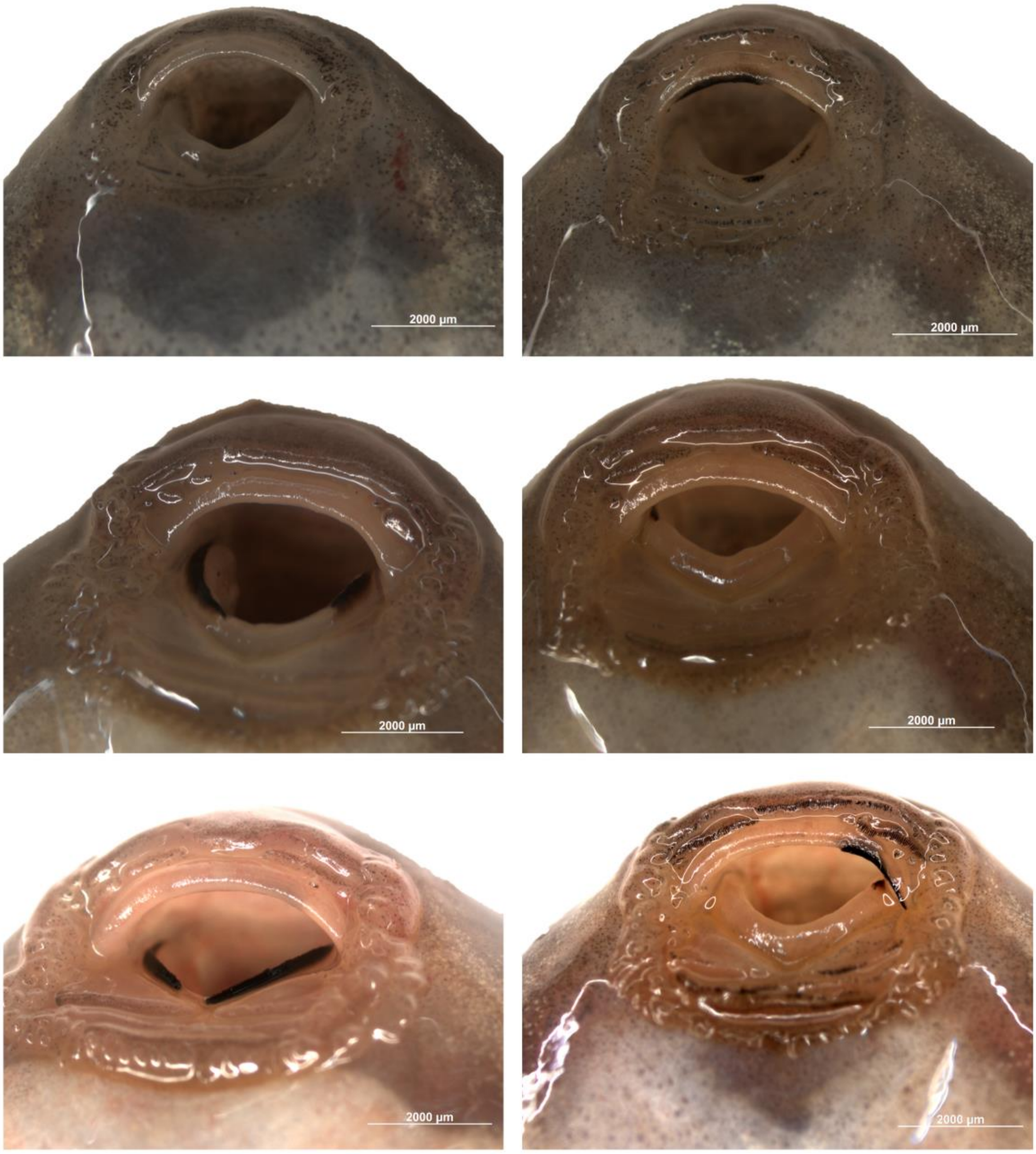

Supplementary Figure S2. Mouthpart dekeratinization in Bd-infected bullfrog tadpoles obtained from a bullfrog farm in the state of São Paulo, Brazil.

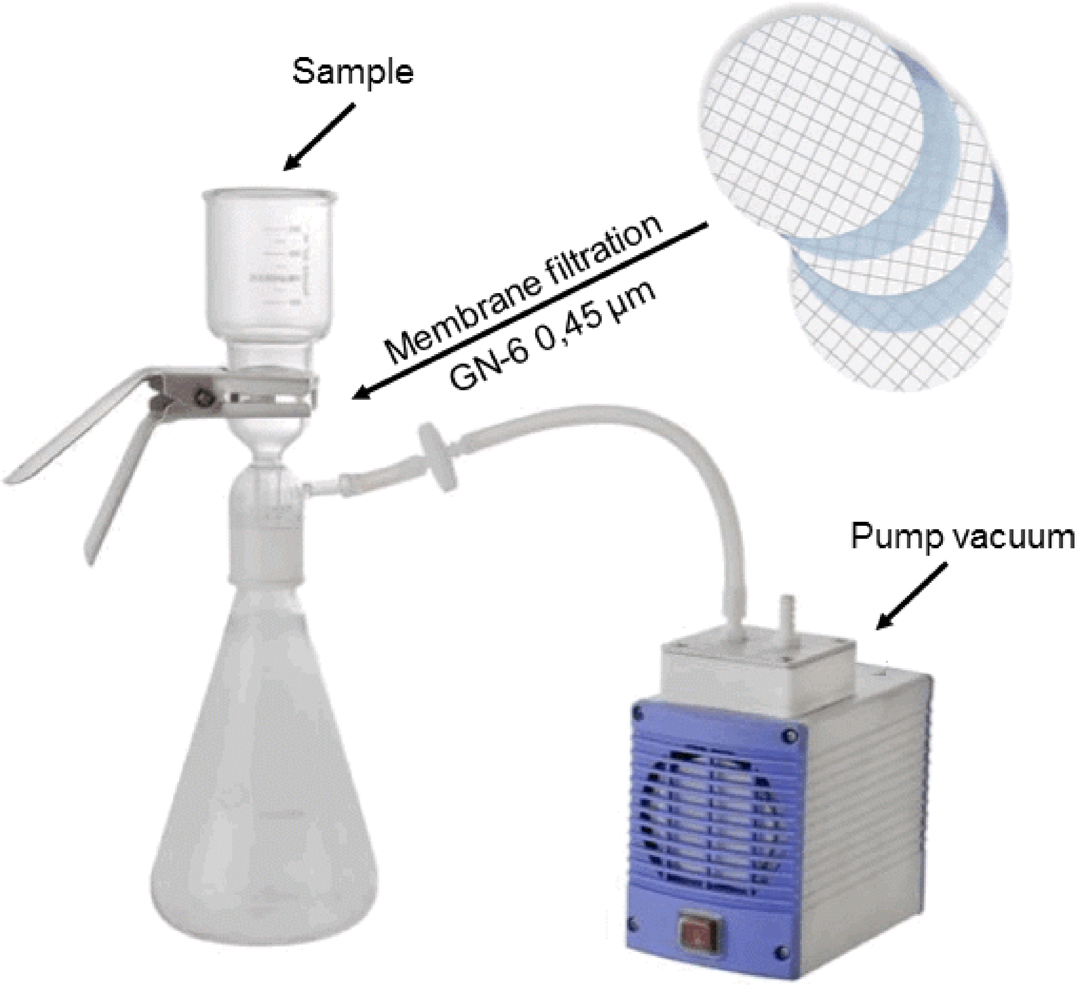

Supplementary Figure S3. Equipment for water filtration procedure for Bd detection.
